# Extracellular Recordings of Patterned Human Pluripotent Stem Cell-Derived Cardiomyocytes on Aligned Fibers

**DOI:** 10.1155/2016/2634013

**Published:** 2016-06-29

**Authors:** Junjun Li, Itsunari Minami, Leqian Yu, Kiyotaka Tsuji, Minako Nakajima, Jing Qiao, Masato Suzuki, Ken Shimono, Norio Nakatsuji, Hitetoshi Kotera, Li Liu, Yong Chen

**Affiliations:** ^1^Institute for Integrated Cell-Material Sciences (WPI-iCeMS), Kyoto University, Yoshida-Ushinomiya-cho, Sakyo-ku, Kyoto 606-8501, Japan; ^2^Department of Micro Engineering, Kyoto University, Katsura, Nishi-ku, Kyoto 615-8540, Japan; ^3^Bio Research Department, Device Research Laboratory, Advanced Research Division, Panasonic Corporation, 3-4 Hikaridai, Seika-cho, Soraku-gun, Kyoto 619-0237, Japan; ^4^Ecole Normale Supérieure, CNRS-ENS-UPMC UMR 8640, 24 Rue Lhomond, 75005 Paris, France

## Abstract

Human induced pluripotent stem cell (hiPSC) derived cardiomyocytes (CMs) hold high potential for use in drug assessment and myocardial regeneration. To create tissue-like constructs of CMs for extracellular monitoring, we placed aligned fibers (AFs) on the surface of a microelectrode array and then seeded hiPSC-CMs for subsequent monitoring for 14 days. As expected, the CMs organized into anisotropic and matured tissue and the extracellular recordings showed reduced premature beating higher signal amplitude and a higher probability of T-wave detection as compared to the culture without fibers. The CMs on the aligned fibers samples also exhibited anisotropic propagation of the field potential. These results therefore suggest that the hiPSC-CMs cultured on AFs can be used more reliably for cell based assays.

## 1. Introduction

Human cardiomyocytes (CMs) can currently be produced by differentiation of human induced pluripotent stem cells (hiPSCs) at high efficiency and high purity [1]. It is then important to form tissue-like CM constructs, rather than nonorganized cellular clusters [2–5], for more reliable drug assessment and myocardial regeneration [2–6]. In heart, cardiac tissues are formed by rod-shaped cardiomyocytes that are aligned in the form of compact and parallel myofibers with coordinated gap junctions. To mimic this organization, CMs have been cultured on patterned surfaces [7, 8], shrunken wrinkles [9], nanofibers [10–12], and biowires [13]. Those techniques promoted the alignment of CMs in either 2D or 3D environment, enhancing the differentiation as well as the maturation of CMs; however, the majority of these studies utilized optical calcium imaging and patch clamp techniques for functional characterization, which are invasive and inconvenient for drug assessment comparing to the extracellular recordings with microelectrode arrays.

In this study, we cultured hiPSC-CMs on aligned fibers (AFs) and followed the cardiac tissue-like construct formation. The fibers were made of polydimethylglutarimide (PMGI), which is biocompatible and can be easily electrospun [14]. Aligned PMGI fibers were produced by electrospinning and then placed by thermal transfer onto a commercial microelectrode array (MEA) to facilitate the electrophysiological monitoring of the tissue-like hiPSC-CMs. As a result, compared with conventional matrices-coated 2D (Flat) and random fiber- (RF-) coated substrate, we observed infiltration of CMs underneath the AF layer and the alignment of distinct sarcomeric bundles. We also observed an increased expression of cardiac maturation markers in CMs cultured on AFs comparing to the control. Accordingly, the extracellular recording of CMs on AFs showed reduced premature beating [15], higher signal amplitude, and higher probability of T-wave recording with respect to the control without fibers. Anisotropic propagation of the field potential was also confirmed, indicating the formation of tissue-like constructs of matured CMs and the reliability of the method for future drug screening and cardiac toxicity studies.

## 2. Methods

### 2.1. Fiber Fabrication and Integration

PMGI solutions with different PMGI concentrations (11%, 13%, 16%, and 19%) (MicroChem, Westborough, MA, USA) dissolved in tetrahydrofuran and cyclopentanone were prepared. For electrospinning, a direct current high-voltage generator (TechDempaz, Tsukuba, Japan) was used to provide a voltage of 8 kV. The solution was loaded into a 1 mL syringe, to which a needle tip of 0.6 mm inner diameter was attached. The positive electrode of the high-voltage power supply was connected to the needle. A grounded rotating drum was used as collector and the speed of the drum was set to 11.4 m/s to obtain the AFs. In the case of random fibers (RFs), the drum was set to 0 m/s. The distance between the tip and the collector was maintained at 12 cm. The humidity was measured to range from 21% to 35% while the temperature was maintained at 25°C. Before spinning, a layer of aluminum foil was attached to the drum. The AFs were first collected by the aluminum foil, which together with the fibers was then peeled off and pressed onto a cover glass (Matsunami Glass Ind. Ltd., Kishiwada, Japan) or an MEA substrate by a Thermo Press Machine (AS ONE, Osaka, Japan). Following removal of the aluminum foil, the fibers were mostly transferred to the substrate.

### 2.2. Differentiation and Culture of hiPSC-CMs

hiPSCs (IMR-90-1) were maintained and differentiated according to the published method [1] following the Kyoto University guidelines. After 30–50 days of differentiation, the floating colonies of CMs were collected and dissociated into single cells by stirring for 1-2 h in protease solution (0.1% collagenase type I, 0.25% trypsin, 1 U/mL DNase I, 116 mM NaCl, 20 mM HEPES, 12.5 mM NaH_2_PO_4_, 5.6 mM glucose, 5.4 mM KCl, and 0.8 mM MgSO_4_ [pH 7.35]). After dispersion, the dissociated cells were filtered using a 40 *μ*m cell strainer (BD Falcon, Bedford, MA, USA) and resuspended in serum-supplemented cardiac differentiation medium (IMDM containing 1% MEM nonessential amino acid solution, 1% penicillin/streptomycin, 2 mM L-glutamine, 0.5 mM L-carnitine (Sigma-Aldrich, St. Louis, MO, USA), 0.001% 2-mercaptoethanol, and 1-2% BSA (Wako, Osaka, Japan), or 0.4% human serum albumin (Sigma-Aldrich)) and plated on AFs and RFs or on 0.1% gelatin-coated flat substrates (Flat) at a density of 1 × 10^6^ cells cm^−2^. The medium was changed to serum-free medium starting from day 2 and replaced every 4 days.

Cryopreserved hiPSC-CMs (iCell) were purchased from Cellular Dynamics International (CDI) (Madison, WI, USA). The cells were thawed in prepared medium (Plating Media, CDI) and plated on 0.1% gelatin (Sigma-Aldrich, USA) coated plates at a density of 0.5 × 10^6^ cells cm^−2^. The medium was changed to culture medium (Maintenance Media, CDI) 2 days later. The culture medium was replaced every two days. The culture was maintained for 7 days and then replated on AF-coated or fibronectin-coated (50 *μ*g/mL, Roche, Roswell, GA, USA) Flat at a density of 1 × 10^6^ cells cm^−2^.

### 2.3. Immunostaining and Imaging

CMs derived from IMR hiPSCs were fixed with 4% paraformaldehyde and were permeabilized in PBS plus 0.5% Triton X-100 for 0.5 h. After blocking in a 5% normal goat serum, 5% normal donkey serum, 3% BSA, and 0.1% Tween 20 in PBS for 16 h at 4°C, the cells were incubated with an *α*-actinin (mouse monoclonal IgG, 1 : 1000; Sigma), troponin T2 (TnT2, mouse monoclonal IgG, 1 : 200, Santa Cruz Biotechnology, Dallas, TX, USA), MLC2v (rabbit polyclonal IgG, 1 : 200; Proteintech Group, Rosemont, IL, USA), or *β*-MHC (mouse MYH7 monoclonal IgM, 1 : 100; Santa Cruz Biotechnology) antibody for 16 h at 4°C. The cells were then washed and incubated with different secondary antibodies diluted in blocking buffer (1 : 1000): DyLight-594 anti-mouse IgG, DyLight 488 anti-mouse IgG, DyLight-594 anti-rabbit IgG, or DyLight-488 anti-mouse IgM (all from Life Technologies) at 25°C for 1 h. Nuclei were visualized by DAPI (Wako).

### 2.4. MEA Recordings

Extracellular recordings from cultured CMs were performed by using a PC-based data acquisition system consisting of the MEA, preamplifiers, filter amplifiers (Multi Channel System, Reutlingen, Germany), data acquisition boards (ADInstruments, Dunedin, New Zealand), and software: Lab chart (ADInstruments) and MATLAB (MathWorks, Natick, MA, USA). The MEA was fabricated on a 50 × 50 mm glass substrate. 60 titanium-nitride electrodes with a diameter of 30 *µ*m and distance of 200 *µ*m were organized in a 1.4 × 1.4 mm matrix in the center of substrate. Cultures were stimulated using one of the electrodes. The MEA with CMs was taken from the incubator and placed on a hotplate controlled by a temperature controller maintained at 37°C. Data were recorded at 20 kHz with 16-bit precision and were digitally filtered by a low pass arithmetic filter to obtain zero phase distortion with a cut-off frequency of 2 kHz. The filtered signal was differentiated digitally to determine the local activation time (LAT) at each electrode, and the activation map was constructed by interpolating the LAT values for the sites between the electrodes within the MEA matrix. The amplitude, QT interval, T-wave recording ratio, and beating rate were determined by analyzing the waveform of the CM field potential. The corrected QT interval (cQT interval) was calculated by normalization to the CM beating rate by using the Fridericia correction formula: $cQT\;\;interval=QT\;\;interval/\sqrt[{3}]{RR\;\;interval}$.

### 2.5. Electron Microscopy

For scanning electron microscopy (SEM), high-resolution images were obtained using a scanning electron microscope (SEM JCM-5000; JEOL Ltd., Tokyo, Japan) operating at 10 kV. A 5 nm thick platinum layer was deposited on the samples by sputtering (MSP 30T; Showa Shinku Device, Sagamihara, Japan). The angular distributions and fiber diameters were evaluated using ImageJ software (National Institutes of Health, Bethesda, MD, USA).

For transmission electron microscopy (TEM), the samples were fixed with 2% glutaraldehyde (Distilled EM Grade, Electron Microscopy Sciences, Hatfield, PA, USA) in NaHCa buffer (100 mM NaCl, 30 mM HEPES, 2 mM CaCl_2_, adjusted to pH 7.4 with NaOH) and then postfixed with 0.25% osmium/0.25% K_4_Fe(CN)_6_, with 1% tannic acid and finally with 50 mM uranyl acetate. The samples were then washed, dehydrated in a series of ethanol solutions, and embedded in TABA EPON 812 resin (TAAB Laboratories Equipment Ltd., Reading, UK). After polymerization at 65°C, ultra-thin sections (60–100 nm) were cut vertical to the PMGI fiber orientation using an ultramicrotome (Leica FC6, Vienna, Austria). The sections were then mounted on EM grids, stained with lead citrate, and observed by TEM (JEOL JEM1400).

### 2.6. Cell Attachment Assay

IMR hiPSC-CMs were seeded on fibers or other substrates at a density of 10^6^ cells cm^−2^ for 5 h, and after the samples were rinsed twice with PBS, the adherent cells were harvested and counted. The cell attachment rate was evaluated using the following equation: cell attachment rate (%) = number of adhered cells × 100/number of seeded cells.

### 2.7. Statistical Analysis

All quantitative data are presented as the means ± standard error of the means. The difference between two groups was analyzed by Student's *t*-test and *P* < 0.05 was considered statically significant.

## 3. Results and Discussion

### 3.1. Preparation of Aligned PMGI Fibers

**Figure 1:**
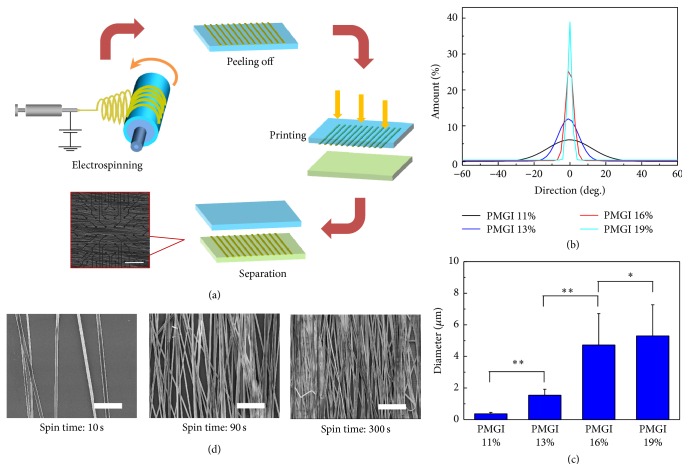
Preparation of aligned PMGI fibers. (a) Schematic of electrospinning and transfer of electrospun nanofibers to the surface of a microelectrode array. The scale bar = 50 *μ*m. (b) Angular distributions and (c) diameter of fibers generated from PMGI of different concentrations (%). (d) Scanning electron microscopy images of 16% PMGI fibers electrospun for 10, 90, and 300 s. The scale bar = 50 *μ*m (means ± s.e., *n* = 3, ^*∗*^ *P* < 0.05, ^*∗∗*^ *P* < 0.01).

Electrospun fibers have been often used to guide the orientation of various types of cells [16]. Here, aligned PMGI fibers were obtained by electrospinning with a rotating drum (Figure 1(a)). The alignment and diameter of fibers could be controlled by changing the concentration of PMGI (Figures 1(b) and 1(c)). The 19% and 16% PMGI fibers demonstrated the best alignment compared to those from lower concentration PMGI. However, at the highest concentration (19%) the PMGI solution exhibits a higher viscosity, resulting in a fiber diameter larger than 5 *μ*m that made transfer onto the MEA surface difficult. In contrast, the 16% PMGI fibers could be easily transferred to the MEA surface owing to the relatively smaller fiber diameters. The density of the fiber sheets could be manipulated by varying the spinning time (Figure 1(d)). Typically, we obtained fibers ranging from a sparse distribution 10 s spinning to a thick fiber layer after 300 s spinning, which might prohibit the infiltration of CMs and impede the contact with the electrodes underneath the fibers. Therefore, 90 s electrospun fibers were chosen for CM culture. The as-spun PMGI fibers were then transferred to the surface of the MEA, which enabled the extracellular recording of CM activities.

### 3.2. CMs Cultured on the PMGI Fibrous Substrate

**Figure 2:**
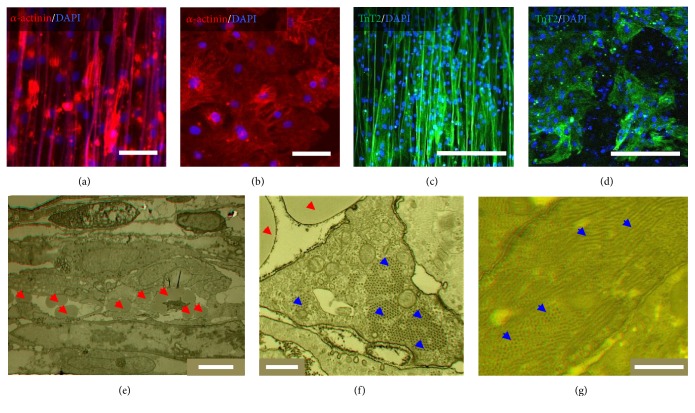
IMR hiPS-cardiomyocytes (CMs) on PMGI fibers. (a–d) Fluorescence image of CMs cultured for 14 days on aligned fibers (AFs) and gelatin-coated flat substrates (Flat). (a, b) *α*-actinin is labeled in red and (c-d) TnT2 in green. The color on fiber is due to autofluorescence of PMGI. The scale bar in (a, b) = 50 *μ*m. The scale bar in (c, d) = 200 *μ*m. (e) Transmission electron microcopy images of CMs cultured for 14 days on AFs. The red arrows mark the fibers. The scale bar = 5 *μ*m. (f, g) Higher magnitude TEM images of CMs cultured for 14 days on AFs (f) and Flat (g). The red and blue arrows mark the fibers and the sarcomeric bundles, respectively. The scale bar = 500 nm.

The IMR hiPSC-CMs were seeded on PMGI fibers and gelatin-coated Flat for attachment tests. At 5 h after seeding, the samples were rinsed with PBS to remove the unattached cells. A significant difference of CM attachment was observed between the three types of substrates, which suggested the reliability of using PMGI fibers (Supplementary Figure  1 in Supplementary Material available online at http://dx.doi.org/10.1155/2016/2634013). Next, immunocytochemical analysis was carried out after seeding CMs on different substrates at day 14 (Figures 2(a)–2(d)). As shown in Figure 2(a), CMs on AF showed elongated shape and preferential orientation in the direction of the AFs. The alignment of the PMGI fibers clearly influenced the orientation of sarcomeric *α*-actinin in the CMs. When the cells were cultured on RF and Flat, the *α*-actinin filaments appeared disordered and scattered in all directions (Figure 2(b) and Supplementary Figure 2(a)). This could also be observed in another cardiomyocyte specific marker expression, TnT2 (Figures 2(c) and 2(d)).

Previously, scaffolds generated by electrospinning tended to have a pore size smaller than 10 *μ*m so that the cells could not easily infiltrate the fibers and form 3D tissue such as the extracellular matrix under* in vivo* conditions [17]. In comparison, in this study, after culture for 14 days, the CMs on the AFs were examined with TEM. Notably, the AFs appeared to have been embraced by the CMs, indicating that the CMs could effectively infiltrate into the area underneath the fibers and organized tissue-like structure with multilayers (Figure 2(e)). Furthermore, the oriented fibers led to the alignment of the huge sarcomeric bundles in the AF samples; these were all vertical to the observing plane (Figure 2(f)). In comparison, randomly arranged sarcomeric bundles, vertical or parallel to the observing plane, could be observed in the RF and Flat sample (Supplementary Figures 2(b) and 2(g)).

### 3.3. AFs Promote the Maturation of hiPSC-CMs

**Figure 3:**
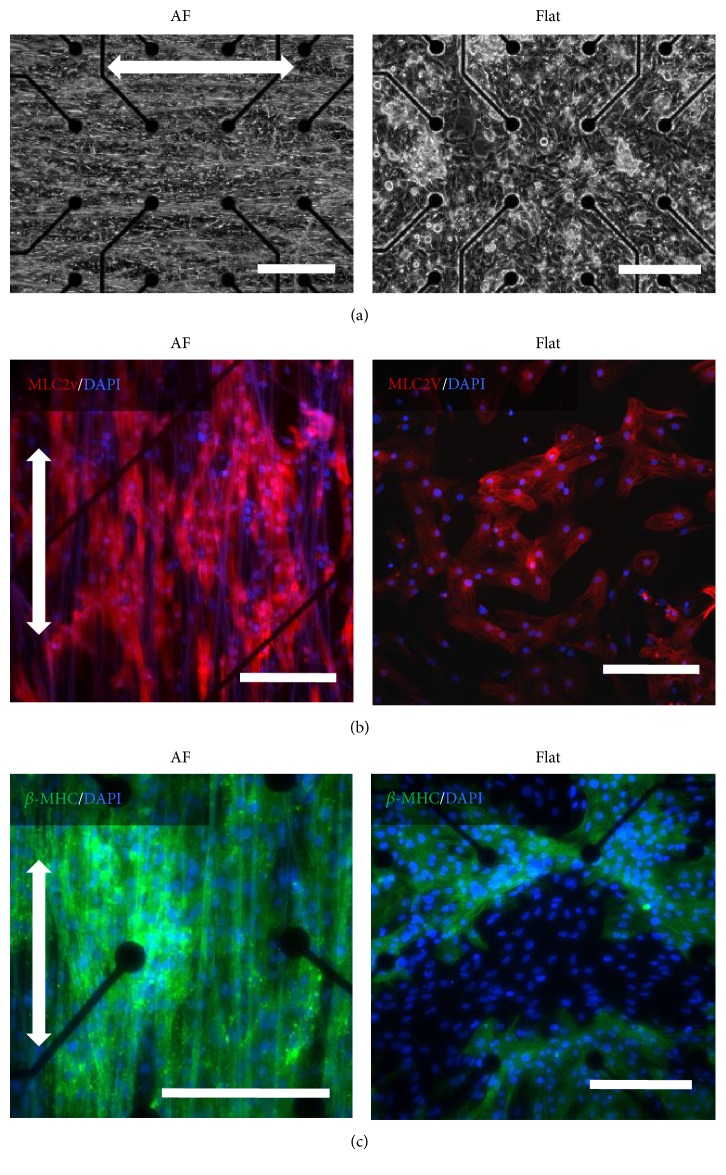
IMR hiPS-cardiomyocytes (CMs) cultured on aligned fiber (AF)-coated and gelatin-coated (Flat) microelectrode arrays (MEA). (a) Phase contrast images of CMs plated on the MEA for 2 days: the scale bar in = 200 *μ*m. (b, c) Fluorescence images of CMs cultured for 14 days on MEA. (b) MLC2v is shown in red, (c) *β*-MHC in green, and DAPI in blue. The scale bar = 200 *μ*m. The color on fiber is due to autofluorescence of PMGI. White arrow marked the fiber orientation.

We next compared the effect of IMR hiPSC-CMs on MEA devices with or without AFs (Figure 3(a)). In addition, CMs were used for extracellular recording. As shown in Figure 3(a), CMs on AF-coated MEA exhibited elongated shapes and more homogeneous morphology than those on the RF and Flat sample. Notably, immunostaining for cardiac tissue-specific markers indicated that significantly more cells were positive in AF samples for MLC2v (Figure 3(b) and Supplementary Figure 2(c)), a marker related to ventricular structures, and *β*-MHC (Figure 3(c) and Supplementary Figure 2(d)), a cardiac maturity marker correlated with contractile velocity [18]. These results indicated the efficiency of AFs in mediating CM organization and maturation than the RF and Flat control.

### 3.4. Electrophysiological Profile of CMs on the PMGI Fibrous Substrate

CMs are electrically active cells. Extracellular recording of spontaneous/stimulated electrical activity in contracting CMs has been shown to enable the assessment of the electrophysiological features of the CMs (Supplementary Figure 3) [19]. Right now, the researches on hiPSC-CMs-based drug assessment are all utilizing the MEA with fibronectin-coated, Matrigel-coated, or gelatin-coated surface [5, 15, 20, 21], and the CMs on RF-coated substrate showed no better organization or maturation than those in Flat sample despite of their enhanced attachment on RFs; we thus chose the Flat as the control in the following electrophysiology assessment.

**Figure 4:**
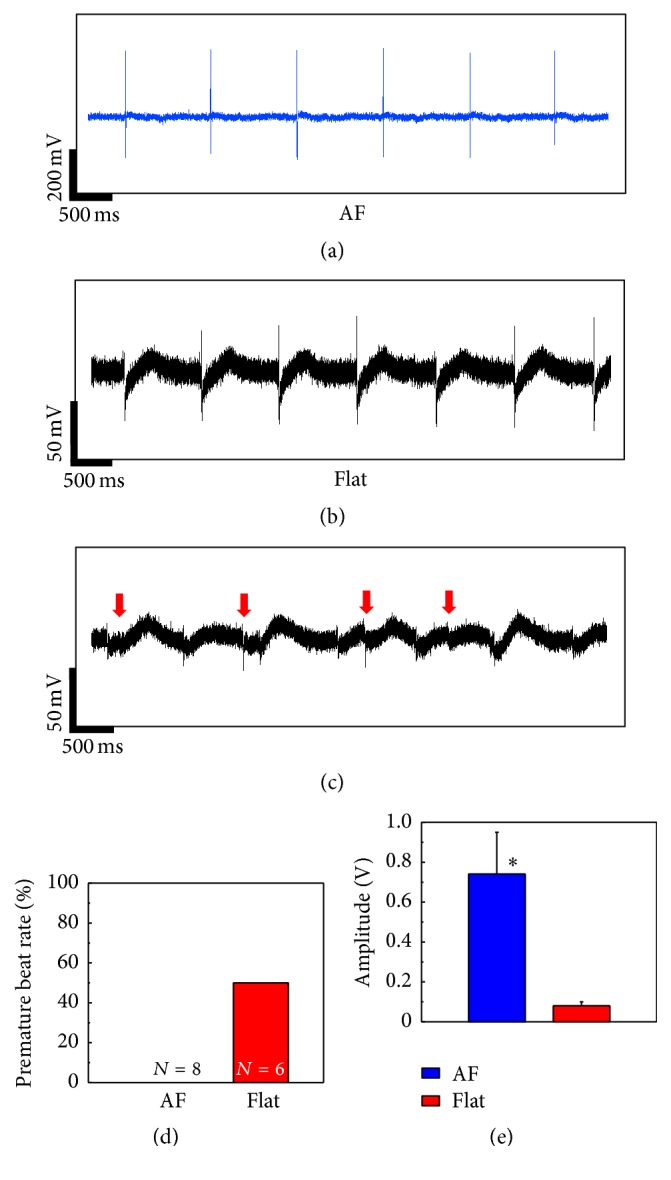
Extracellular recording of IMR hiPS-cardiomyocytes (CMs) by MEA at day 6. Spontaneous beatings of CMs on aligned fiber- (AF-) covered (a) and gelatin-coated (Flat) MEA (b) at day 6. (c) Premature beating recorded on Flat samples at day 6. The red arrows mark the representative beating of the premature beating. (d) Premature beating rate of samples at day 6. *N* is the number of the respective samples. (e) Amplitude of field potential recorded on CMs at day 6 (means ± s.e., *n* = 3, ^*∗*^ *P* < 0.05).

**Figure 5:**
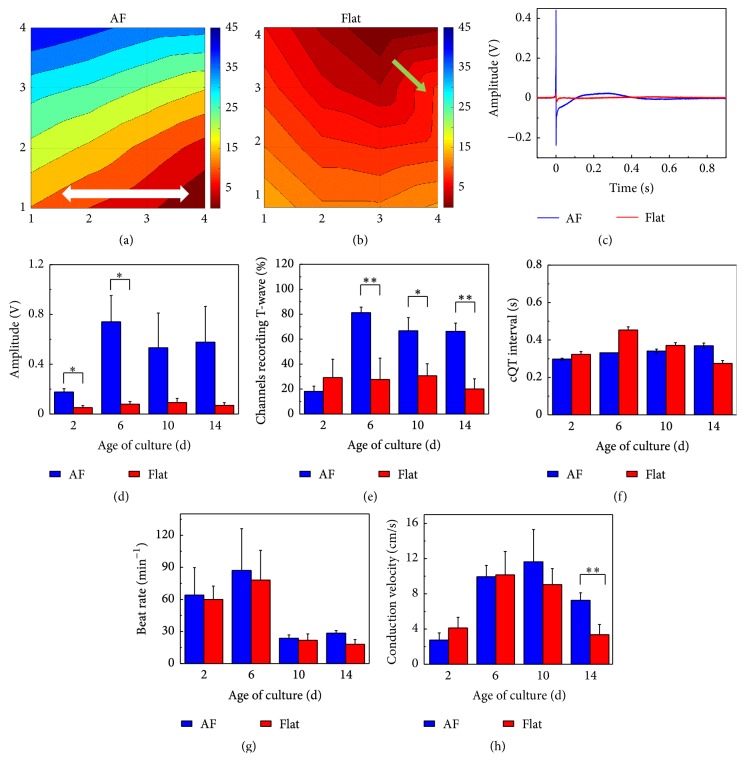
Electrical characterization of IMR hiPS-cardiomyocytes (CMs). (a-b) Activation maps showing the propagation of contraction on day 6. The white arrow marks the fiber orientation and the green arrow marks the delay area. (c) Field potentials (FPs) of CMs cultured on the two types of substrates at day 6. (d) Amplitude of FP at different culture times (means ± s.e., *n* = 3, ^*∗*^ *P* < 0.05). (e) The ratio of the channels recording the T-wave (means ± s.e., *n* = 3, ^*∗*^ *P* < 0.05, ^*∗∗*^ *P* < 0.01). (f) cQT intervals recorded from CMs at different culture times (means ± s.e., *n* = 3). (g) CM beating rate at different culture times (means ± s.e., *n* = 3). (h) Conduction velocity of spontaneous contraction propagation at different culture times (means ± s.e., *n* = 3, ^*∗∗*^ *P* < 0.01).

In addition to the observation of spontaneous beating at day 6 (Figures 4(a) and 4(b)), we recorded premature beats within some of the Flat samples (Figures 4(c) and 4(d)) indicating lower synchronization among the IMR hiPSC-CMs in the Flat samples. In contrast, no premature beats were recorded with AF samples, indicating reliable intercellular connectivity and coupling of CMs in the tissue-like constructs, which can be also confirmed by the dramatically higher amplitude of recorded signal on day 6 (Figure 4(e)). The differences in connectivity and coupling between the two types of samples could be seen more clearly in the activation maps drawn based on the spontaneous contractility (Figures 5(a) and 5(b)). Here, CMs in the Flat samples showed a regional conduction delay but no such effect was observed in the AF sample. Furthermore, we observed a higher amplitude of field potential (FP) from the CMs plated on AFs compared to those on Flat during 14 days, which indicated a better cell attachment on AF-coated MEA surfaces (Figures 5(c) and 5(d)). The same result was confirmed by using cultured commercial CMs (iCell) on two types of substrates (Supplementary Figures 4(a) and 4(b)).

Moreover, we evaluated the percentage of the channels that had a recorded T-wave and identified a much higher recording ratio with the AF samples compared to the Flat samples (Figure 5(e) and Supplementary Figure 4(c)). Since the T-wave recording is important for determining the QT interval variation during drug assessment, our results suggest not only a better cellular attachment but also more robust signal readout with the CMs cultured on AF-coated MEA surfaces. On the other hand, we noted higher T-wave amplitude of IMR hiPSC-CMs than of iCell (Figure 5(c) and Supplementary Figure 4(a)). The cQT interval showed no obvious difference between IMR hiPSC-CMs in AF and Flat samples (Figure 5(f)). However, the IMR hiPSC-CMs showed dramatically shorter QT intervals than the iCell (Figure 5(c) and Supplementary Figure 4(a)), and after applying Fridericia correction, the cQT intervals showed slight difference between two types of cells (Figure 5(f) and Supplementary Figure 4(d)). The iCell beats faster in Flat samples than those in AF samples on day 6 and day 10 while there are no such differences in IMR hiPSC-CMs (Supplementary Figures 4(e) and 5(g)). Moreover, our data may indicate the best timing for carrying out CMs based drug test which may be at around 6 days, similar to the recommended timing [22]. These data indicated intrinsic differences between IMR hiPSC-CMs and the commercial iCell due to different differentiation methods. The conduction velocity of the spontaneous contraction showed no dramatic difference between AF and Flat samples (Figure 5(h), Supplementary Figure 4(f)).

**Figure 6:**
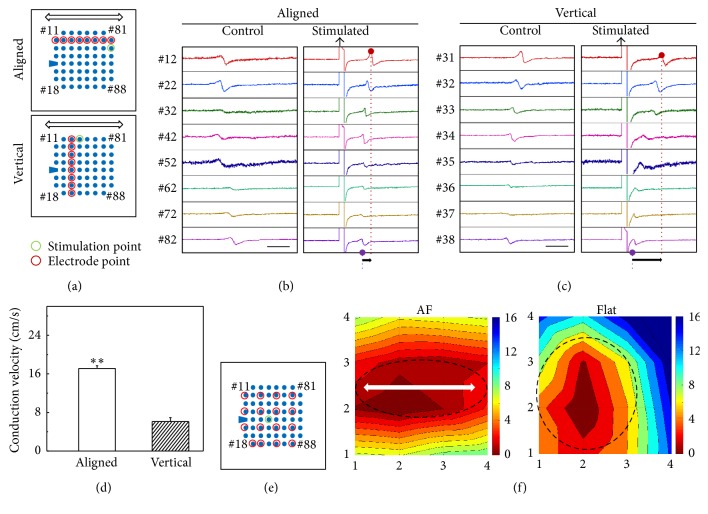
Propagation of the field potential of IMR hiPS-cardiomyocytes. (a) Electrode arrangement for recording and stimulation. The AF orientation is marked by arrow. (b, c) Time course of the field potential along (b) and perpendicular (c) to the fiber direction. The scale bar = 0.2 s. The black arrows mark the starting point of stimulation. (d) Conduction velocity of the stimulated contraction in different directions (means ± s.e., *n* = 3, ^*∗∗*^ *P* < 0.01). (e) Electrode arrangement for activation map analysis. (f) Activation maps showing the propagation of stimulated contraction at day 14. The color bar from red to blue is linearly divided from 0 ms to 16 ms. The arrow marks the AF orientation. The encircled line marks the shape of the isochrones of the propagation.

The electrical stimulation has been used to pace hiPSC-CMs [23]; here we applied stimulation (±700 mV, 5 ms duration) (Figure 6(a)) to quantitatively compare the propagation speed in different directions; for this experiment, the stimulation and recording electrodes were arranged as shown in Figure 6(a). The time course of the field potential after stimulation showed a uniformly propagation and the field potential propagated from one electrode to another along a pathway originating from the location close to the stimulation electrodes (Figures 6(b) and 6(c)). The propagation speed along the fiber direction was more than twofold larger than that perpendicular to the fiber direction (Figure 6(d)), similar to the observation with other types of anisotropic substrates [24, 25]. Furthermore, the activation map obtained by 4 × 4 electrode array arrangement (Figure 6(e)) showed elliptical isochrones with the AF covered samples but an isotropic propagation in the Flat samples (Figure 6(f)). In summary, the AF-coated MEA promoted anisotropic cellular organization and anisotropic propagation of the field potential, enhancing the extracellular recording of the cultured CMs. Such an integrated platform thus holds high potential for future studies.

## 4. Conclusion

Electrospun AFs were placed on the surface of a commercial MEA. hiPSC-CMs could be cultured thereon, which resulted in tissue-like CM constructs. The CMs in such a tissue-like constructs were elongated, compact, and matured, showing reduced premature beating, higher signal amplitude, and higher probability of T-wave recording compared with those cultured on the flat substrate without fibers. We therefore confirmed the robustness of AF-coated MEA for the creation of tissue-like constructs of hiPSC-CMs and their reliability for extracellular recording.

## Supplementary Material

Supplementary Figure 1: IMR hiPS-cardiomyocytes attachment rate on different substrates.
Supplementary Figure 2: Cardiomyocytes (CMs) cultured on random fiber (RFs).
Supplementary Figure 3: Data acquisition by MEA and construction of the activation map of cardiomyocytes (CMs).
Supplementary Figure 4: Electrical characterization of the commercial cardiomyocytes (CMs, iCell) on the MEA system.
