# Supplementary material for: Extracellular Recordings of Patterned Human Pluripotent Stem Cell-Derived Cardiomyocytes on Aligned Fibers
Source: Stem Cells Int. 2016 Jun 29;2016:2634013. doi: 10.1155/2016/2634013 (PMC4942673; doi:10.1155/2016/2634013)
Supplement: Supplementary file 1 — Supplementary Figure 1: IMR hiPS-cardiomyocytes attachment rate on different substrates. Supplementary Figure 2: Cardiomyocytes (CMs) cultured on random fiber (RFs). Supplementary Figure 3: Data acquisition by MEA and construction of the activation map of cardiomyocytes (CMs). Supplementary Figure 4: Electrical characterization of the commercial cardiomyocytes (CMs, iCell) on the MEA system. [file 2634013.f1.pdf]

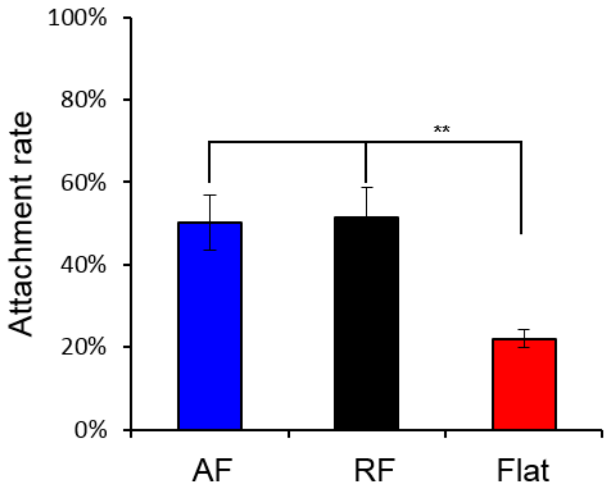

Supplementary Figure 1: IMR hiPS-cardiomyocytes attachment rate on different substrates (means ± s.e., n = 3, ***P*<0.01).

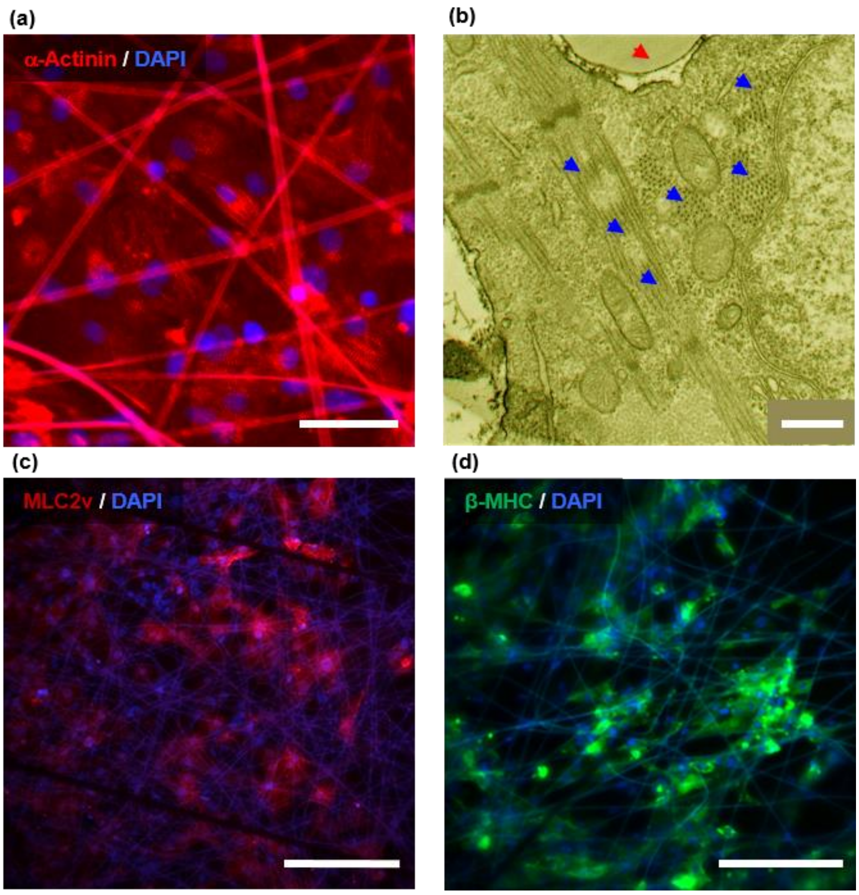

Supplementary Figure 2: Cardiomyocytes (CMs) cultured on random fiber (RFs). (a, c, d) Fluorescence image of CMs cultured 14 day on random fibers (RFs). a-Actinin (a) is in red, the scale bar = 50 μm. (b) The transmission electron microcopy (TEM) images of CMs cultured 14 day on RFs. The red and blue arrows marked the fibers and sarcomeric bundles respectively. The scale bar = 500 nm. (c) MLC2v is in red, the scale bar = 200 μm. (d) β-MHC is in green. The scale bar = 200 μm.

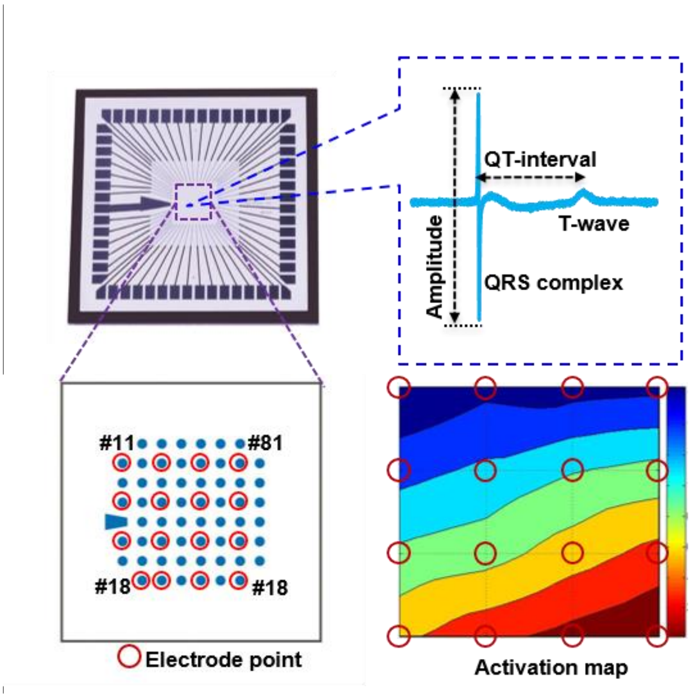

Supplementary Figure 3: Data acquisition by MEA and construction of the activation map of cardiomyocytes (CMs). MEA chip and electrode arrays on which CMs were seeded. The encircled image is a representative electrogram of one field potential recorded from CMs, showing the parameters analyzed. The electrodes marked with red circles were used for recording. The numbers in the four corners are markers of electrode location. Typical activation maps were constructed from the local activation time at each of the 16 electrodes.

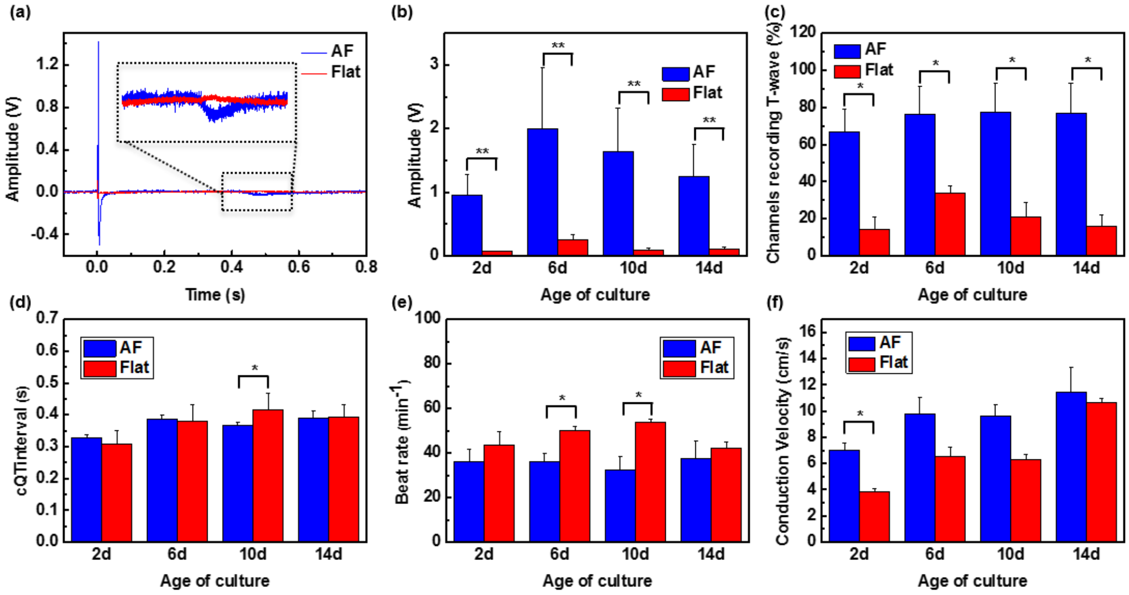

Supplementary Figure 4: Electrical characterization of the commercial cardiomyocytes (CMs, iCell) on the MEA system. (a) Field potentials (FPs) of CMs cultured on different substrates at day 6. The enlarged image shows the T-wave. (b) Amplitude of FP at different culture times. (means ± s.e., n = 3, **P<0.01). (c) The ratio of channels recording the T-wave (means ± s.e., n = 3, *P<0.05) (d) cQT intervals recorded from CMs at different culture times. (means ± s.e., n = 3). (e) CM beating rate at different culture times. (means ± s.e., n = 3). (f) Conduction velocity of spontaneous contraction propagation at different culture times. (means ± s.e., n = 3, *P<0.05).
